# Implications of shorter sampling durations on the analysis of municipal solid waste generation and composition

**DOI:** 10.1007/s10163-025-02401-6

**Published:** 2025-10-30

**Authors:** Emenda Sembiring, Attar Hikmahtiar Ramadan, Muh Farid

**Affiliations:** Air and Waste Research Group, Faculty of Civil and Environmental Engineering, Bandung Institute of Technology, Bandung, 40132 Indonesia

**Keywords:** Waste sampling duration, Household waste analysis, Seasonal variation, Statistical analysis, Cost-effective sampling, Municipal solid waste

## Abstract

Collecting samples of waste generation is essential for designing effective waste management strategies. Conducting waste generation sampling over a week or an 8-day period needs substantial demands on resources. The resource-intensive nature of extended sampling periods is notable. Shortening sampling days is beneficial and perhaps can also be cost effective. Cost-effective means reducing resource demands while maintaining the accuracy and reliability of waste generation data. The solid waste generation sampling data from two sub-districts both in *Banyuwangi* Regency and in *Jembrana* Regency, Indonesia were used for data source. Normality test and ANOVA (analysis of variance), followed by Tukey's post hoc analysis was utilized to analyze the variability among sampling duration, weekdays and weekend, and seasonal change. The generation of household wastes appears consistent in patterns over 4 or 8 days, irrespective of the sampling days falling on weekdays or weekends. When comparing the rainy and dry seasons, it was observed that only one sub-district in *Muncar* exhibited a statistically significant difference in waste generation. The remaining locations demonstrate no substantial statistical variation between the two seasons. This suggests a level of uniformity in waste generation across different time periods and seasonal condition, with Muncar being the sole exception.

**Supplementary Information:**

The online version contains supplementary material available at 10.1007/s10163-025-02401-6.

## Introduction

Municipal solid waste (MSW) generation and management is a global concern, involving a cycle composed of several interrelated stages, such as production and consumption cycle, starting from manufacture stage, distribution stage, and consumption stage and post consumption stage [7]. Waste generation sampling is crucial for waste management planning and assessing its environmental impact. Understanding waste management is vital for achieving circularity and sustainability especially for recovering materials effectively. Waste generation data are also essential for making informed decisions for managing waste effectively. Availability of reliable and continuous data is one of the important keys [18]. Different methods apply for different waste generators; for examples, waste samples collecting from households or from commercial areas [13, 25, 30, 33] or directly from waste collection vehicles [24]. These methods help gather accurate data on what types of waste are generated and where. 

Currently, most common methods of waste generation sampling typically were conducted at least one week which aimed at comprehensively capturing waste generation patterns over a full week, encompassing both weekdays and weekends [4, 9, 25, 30, 33] (Table 1). This approach is widely adopted across waste management studies due to its effectiveness in providing a representative overview of waste generation behaviors within various temporal contexts. The sampling period is lasted for a full week [20], [22]. However, some sampling activities take more times such as waste  characterization (weighing, sorting, and other treatments/analyses) sampling in Sri Lanka and Central Nigeria was conducted for 11 days [29].

**Table 1:** Variability of sampling duration

Sampling duration (Days)	Reference
3	[32], [4]
5	[5]
5–7	[36], [37]
7	[6], [11], [17], [13], [3], [22], [20], [25]
8	[12], [33]
11	[29]
14	[10]
1–14 (median 5 days)	[27]
30	[31]

Conducting waste generation sampling over a week or an 8-day period can incur significant resource demands, including  logistical challenges and the resource-intensive nature associated with prolonged sampling periods. These challenges include the need for sustained manpower, material resources for collecting samples and waste characterization. Despite these challenges, the 8-day sampling approach remains prevalent in waste management research due to its ability to capture variability in waste generation across different days of the week, thereby enhancing the robustness of data analysis and decision-making processes. Nonetheless, ongoing efforts are warranted to explore alternative sampling methodologies and innovative technologies that can mitigate resource demands while ensuring the accuracy and reliability of waste generation data. Sometimes, waste survey and sampling are conducted as quickly as possible to reduce the impact of the rapidly changing political and environmental conditions [10].

In this paper, we conducted an analysis on the significant difference in waste generation patterns by comparing 4 days of sampling duration with 8 days of sampling duration, comparing weekend sampling time with weekdays sampling time, and also comparing rainy season with dry season sampling period. Season variations (rainy and dry seasons) can affect waste generation patterns [28]. Utilizing statistical methods, we aimed to elucidate any notable differences arising from varying sampling durations. By employing statistical techniques, such as analysis of variance (ANOVA), we sought to discern significant differences in waste generation patterns across varying sampling durations. Our findings contribute to the methodological refinement of waste generation studies and inform decision-making processes in waste management strategies within a limited time. Furthermore, our research underscores the importance of considering sampling duration as a critical factor in designing waste generation sampling protocols, ensuring the accuracy and reliability of data for informed decision-making in waste management practices.

## Methods

### Waste generation sampling

In Indonesia, most of the time, a waste characterization survey for waste generation and waste composition  are following the guidelines in SNI 19-3964-1994 (Indonesian Standard for waste generation and composition) [12]. In SNI 19-3964-1994, the waste generation sampling procedure measure waste generation and composition in both residential and non residential area for eight consecutive days. Similarly, other sampling method outlines sampling procedure and measure waste generation and composition in both residential and non-residential areas [5]. These sampling duration and composition are similar to the other international methods of solid waste sampling [4, 25, 33].

Waste sampling for this study was conducted twice in each   regency from October 2022 to November 2023 to account for Indonesia's distinct seasons: rainy and dry seasons. For each regency, the activities—from preparation and permission to conduct the surveys until final day of survey  was spent  — approximately two weeks. This study focused on two regencies, *Jembrana* and *Banyuwangi*, with two sub-districts selected in each: *Jembrana* and *Negara* in *Jembrana* Regency, and *Banyuwangi* and *Muncar* in *Banyuwangi* Regency. Each location had a team of four assistants from the university and four trained local helpers. The local helpers supported various activities, including surveys, sampling, administering questionnaires, and waste composition analysis. To ensure accuracy and consistency, local helpers operated under the supervision and guidance of university assistants, who collaborated closely throughout all sampling activities. Furthermore, during composition analysis, local helpers and university assistants worked together, with local helpers receiving continuous supervision from the start to the end of each day’s sampling. This thorough oversight ensured high-quality and reliable sampling results.

The household waste samples (units) were identified by codes, indicating income level for domestic samples. Waste was collected, weighed using digital scales, and documented. Waste generation data were calculated per unit and multiplied by the number of units to determine total generation. In total, we collected approximately 400 samples from the households divided into 3 economic categories: high income, middle income, and low income. In this study, we also analyzed the waste composition for each sample. The categories of samples are divided into several distinct types: organics (food wastes and garden wastes), wood, paper, metal, rubber and leather, textile, glass/mineral, plastics, hazardous waste, electronic waste, bulky waste, and others. Each sample is meticulously sorted into these categories to ensure a comprehensive understanding of the waste composition. Sample information is provided in Table 2. These study areas were chosen to represent a diverse range of characteristics, including urban and rural settings. By selecting Banyuwangi (East Java) and Jembrana (Bali) regencies as the study areas, our research captures diverse waste generation patterns across non-metropolitan regions that reflect varying socio-economic, occupational, and lifestyle conditions. Banyuwangi, with a population of over 1.7 million, is characterized by a mix of rural and semi-urban areas where livelihoods are dominated by agriculture, fisheries, small-scale industries, and tourism. In contrast, Jembrana, with a smaller population of around 320,000, represents a predominantly rural Balinese context where agriculture, livestock, and traditional occupations prevail. Both regions exhibit informal and community-based waste management systems though Banyuwangi has more developed infrastructures in urban zones. While Banyuwangi’s coastal communities show a higher plastic use due to fishing activities, Jembrana’s cultural practices contribute to a higher proportion of degradable waste. These differences and similarities make the two regencies both comparable and representative of broader waste generation dynamics in non-metropolitan Indonesian settings.

**Table 2:** Sample size and amount of waste collected for analysis

No	Regency	Sub-district (Collection site)	Number of samples used (Household)		Total waste collected for analysis (kg)	
			Dry season	Rainy season	Dry season	Rainy season
1	*Banyuwangi*	*Banyuwangi* (*Banyuwangi* Waste Bank)	50	41	506.37	436.84
		*Muncar* (*TPS3R Tembokrejo, Muncar*	46	38	382.24	630.69
2	*Jembrana*	*Jembrana*	35	28	615.27	322.56
		*Negara* *(TPST Peh, Jembrana*	36	37	496.50	614.05

Furthermore, this study includes a questionnaire to administer information about the occupational background of each respondents. All respondents were asked questions covering, their current job title, type of employment, and sector of work. The aim of collecting this information is to examine the potential relationship between occupational backgrounds on waste generation dynamics. This will enable a more in-depth analysis of how different occupations may influence or relate to validity of reduce sampling duration.

### Statistical analysis on waste generation and waste composition

In our analysis, we aimed to compare solid waste generation across various segments of the sampling period, which we divided into four distinct durations: first four days, second to sixth days, last four days, weekend, weekdays and the overall eight-day period. Only data sets with a minimum of six days of complete sampling were included in the analysis. Household waste samples that failed to meet this six-day threshold were excluded from consideration. Ultimately, the analysis utilized 77% of the total collected data, ensuring the reliability and completeness of the findings.

To evaluate any statistical differences among these durations, we employed two primary statistical tests: normality test and ANOVA (analysis of variance), complemented by Tukey's post hoc analysis. The normality test was utilized to ascertain whether the data collected for waste generation followed a normal distribution within each segment of the sampling period. This test is crucial as it helps ensure the validity of subsequent statistical analyses, particularly ANOVA. Following the normality test, we conducted ANOVA to compare the means of waste generation across different sampling durations. ANOVA allows the simultaneous comparison of multiple groups, in this case, the four segments of the sampling period, to determine whether there are significant differences in waste generation among them. To further elucidate any significant differences identified by ANOVA, we employed Tukey's post hoc analysis. This analysis is designed to perform pairwise comparisons between all possible combinations of sampling durations, providing insights into which specific durations exhibit statistically significant differences in waste generation.

In the second analysis, we also investigate the statistical differences in waste composition for each sampling period, similar to the waste generation period. For this analysis, we utilize the Friedman test for each waste category as the data are presented as percentages, making it suitable for non-parametric testing. The waste categories used in this analysis are based on the samples category, such as organic, wood, paper, metal, rubber and leather, textile, glass/mineral, plastic, hazardous waste, electronic waste, bulky waste, and others. This approach allows us to determine any significant variations in waste composition over different sampling periods, providing insights into trends and changes in waste generation and composition. By employing the Friedman test, we ensure that the statistical analysis accommodates the nature of our limited number of data.

## Result and discussion

In Indonesia, most of waste generation and waste characterization is conducted in 8 days and in both two seasons dry and wet season [5], [12]. Based on some previous studies, solid waste generation sampling can be conducted within 3–5 days [23]. Other reference, the ASTM stated that sampling can be conducted from 5 to 7 days [4]. This study investigated the feasibility of reducing the sampling duration to just 4 days.

### Comparison of waste generation and composition between consecutive 8-day and 4-day periods, and between weekdays and weekends

To compare the waste generation results, if reducing the sampling period from 8 to 4 days can be significantly different or not, a statistical analysis was conducted. This analysis was conducted across four sub-districts in 2 regencies in Indonesia and two seasonal conditions. In the analysis of statistical difference between sampling durations, through consecutive 4-day variations, the data were analyzed using Tukey’s post hoc analysis. The results presented in Table 3 reveal that for waste generation, no significant differences were noticed between the 4-day and 8-day sampling durations across all sub-districts and no significant differences of waste generation if the sampling was conducted in weekdays or weekends. Consequently, it was concluded that sampling waste generation in Indonesia can be effectively streamlined to a 4-day period, ensuring efficiency without compromising the accuracy of results, irrespective of the type of daily activity. To assess the significance of these findings, the ANOVA analysis was employed, with significance levels set at 0.95 and 0.99.

**Table 3:** Comparison of the statistical differences in waste generation between 8 days, reduced days, and weekends and weekdays of sampling

Sample locations	Mean comparison day							Normality test (*p *value
	(Tukey’s post hoc analysis)							
	8 days	First 4 days	2nd–5th days	3rd–6th days	4th–7th days	Last 4 days	Weekend	
Banyuwangi dry season	0,3689*	0,3516*	0.3404*	0,3426*	0.3366*	0,3558*	0,3746*	< 0.01
Muncar dry season	0,3050*	0,3214*	0.3133*	0,3820*	0.3604*	0,3435*	0,3526*	< 0.01
Jembrana rainy season	0,4359*	0,5636*	0.5936*	0,5299*	0.5497*	0,4829*	0,3233*	< 0.01
Negara rainy season	0,6013*	0,5672*	0.5297*	0,5726*	0.5505*	0,5432*	0,5495*	< 0.01
Banyuwangi rainy season	0,4305*	0,4158*	0.4462*	0,3761*	0.4292*	0,4033*	0,522*	< 0.01
Muncar rainy season	0,5981*	0,4796*	0.4731*	0,4011*	0.4470*	0,4996*	0,6001*	< 0.01
Jembrana dry season	0,5949*	0,5335*	0.5462*	0,5140*	0.5515*	0,5545*	0,5238*	< 0.027
Negara dry season	0,4997*	0,4546*	0.4547*	0,4460*	0.4627*	0,4729*	0,4731*	< 0.01

^*^No significant difference between 4 and 8 days as well as between weekend and weekday of sampling in ANOVA statistical analysis complemented using Tukey’s post hoc analysis (using confidence levels 95% and 99%)

Furthermore, we conducted a comprehensive analysis for waste composition comparison of 8 days of sampling period with 4 days of sampling period as well as comparing weekends with weekdays (Table 4), and the data of the waste composition are provided in the supplementary information. This study covered multiple locations in the Banyuwangi and Jembrana regions during both dry and rainy seasons. No statistically significant differences were noticed in waste composition across the sampling periods. This result consistency aligns with the waste generation result. However, in Negara, during the dry season, a significant difference was detected (*p* = 0.008). This finding suggests variations in waste composition specific to this location, and composition differences occurred in this location because the samples of this location have more office job occupations which leads to differences in composition between weekend and weekdays. This distinct result from Negara underscores the importance of considering local context when analyzing waste composition in each region of sampling.

**Table 4:** Comparison of the statistical differences in waste composition between 8 days, reduced days, and weekends and weekdays of sampling

Sample locations	Friedman analysis (*p* value Result)	Notes
Banyuwangi dry season	0.393	No significant differences
Muncar dry season	0.836	No significant differences
Jembrana rainy season	0.486	No significant differences
Negara rainy season	0.910	No significant differences
Banyuwangi rainy season	0.846	No significant differences
Muncar rainy season	0.784	No significant differences
Jembrana dry season	0.232	No significant differences
Negara dry season	0.008*	There is a significant differences

^*^There is a significant difference in seasons in ANOVA statistical analysis complemented with Friedman analysis (using confidence level 95%)

This result is the difference with the previous study in the United States which showed a higher rate of residential waste disposal observed during weekends compared to weekdays [28]. The other finding also found a similar trend in Botswana [21]. This finding suggests that there may be distinct patterns in waste disposal behavior among residents, in weekends, a surge in waste generation, possibly due to increased household activities or social gatherings. The variation in waste generation rates is attributed to a combination of factors, namely local economy, cultural practices, urbanization, and individual behaviors within each location [35]. The reference to the economy suggests that the level of economic development in each area may play a role in determining waste generation patterns. Regions with higher levels of economic activity and consumerism may produce more wastes due to increased consumption and disposal of goods. Cultural influences are also cited as a contributing factor. Different cultural practices and attitudes toward consumption, recycling, and waste disposal can significantly impact the amount of waste generated within a community [34]. For example, communities with strong traditions of reuse and recycling may produce less waste compared to those where disposal is the prevailing norm. Individual behaviors further contribute to the observed differences in waste generation. Factors, such as household size, lifestyle choices, and awareness of waste management practices, can influence the amount of waste generated by each person on a daily basis [19].

**Fig. 1:**
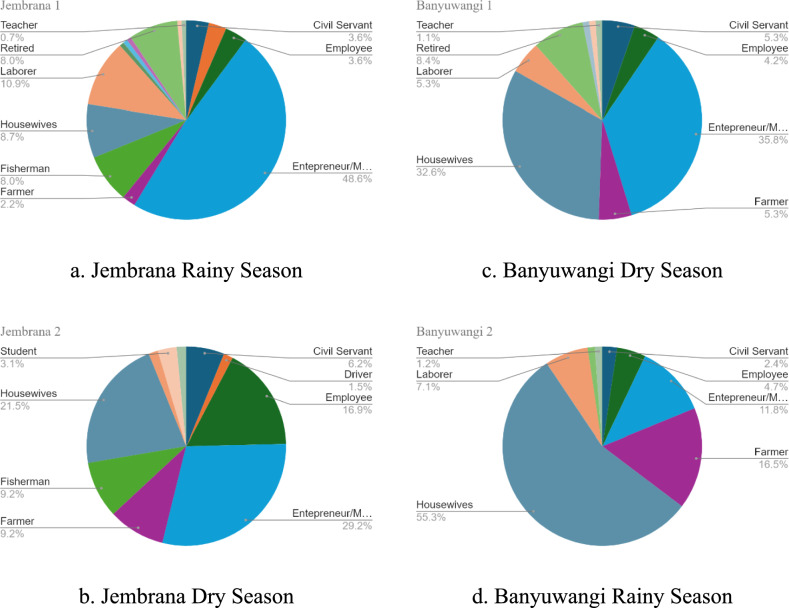
Respondent employment data in Banyuwangi and Jembrana

According to the Fig. 1, the populations in both sampling locations primarily consist of individuals who do not follow the typical office job schedule of five days a week. The findings indicate that the majority of the samples are entrepreneurs, merchants, housewives, and farmers. Employees constitute a small percentage of the population, with less than 10% being engaged in office-based employment. Based on the demographic characteristics in these locations, it is true that sample locations are not metropolitan areas but rather rural municipalities. Furthermore, a significant portion of the population involved in non-office-based occupations, such as agriculture, entrepreneurship, and trade. This indicates a distinct socio-economic profile characterized by a reliance on non-traditional employment sectors. Therefore, it is possible that the activities of these communities do not vary significantly between weekdays and weekends. This statement is also supported by a research which was conducted by [1], which states that waste generation also can be influenced by the variations in employment and livelihood patterns across the area. As a result, the waste generation is not likely to be significantly different whether the samples were taken in 4 days or 8 days.

This suggests that a reduction in the sampling duration method would be suitable for locations that are more rural or that have populations where the majority of people are not engaged in office jobs; for examples, farmers or merchant. In such areas, the daily activities and consequently the waste generation patterns are more consistent throughout the week (weekdays or weekend), making shorter sampling periods an efficient method for waste assessment. Conversely, if the population predominantly occupies office jobs, it is advisable to sample across the entire week to 8 days, including both weekdays and weekends. If the sampling period is less than seven days, it should still encompass both weekdays and weekends to ensure representativeness.

### Comparison among seasons of sampling

To manage MSW adequately, it is essential to know the variables of seasonal conditions as precisely as possible in a specific geographic area. The seasonal fluctuation in the composition of municipal solid waste (MSW) significantly impacts the data quality of organic, recyclable and non-recyclable wastes, along with other contributing factors. Several studies, including those conducted by [2, 11, 15, 16], have specifically investigated the seasonal fluctuations in municipal solid waste (MSW). Their findings uniformly indicate that the physicochemical properties and composition of MSW exhibit noticeable variations ranging from minimal to considerable across different seasons. Based on the findings of an analysis conducted on waste sampling, the range of waste generation in 4 locations of sub-district in Indonesia ranges from 0.3 to 0.6 kg/person/day. The waste generation is similar to the previous studies in *Banyuwangi*, which is around 0.37 [8]. The waste generation rate for this result falls below the average of major cities in Indonesia, which is around 0.69 kg per person per day [26].

The seasonal changes in MSW generation and composition are usually discussed through the identification of main factors affecting these changes. Significant disparities were observed among various seasons concerning fractions, such as organic matter, paper, and metal. An increase in solid waste generation and alterations in composition, particularly a rise in packaging waste, during the summer months on Crete Island is one of the examples [14]. Similarly, MSW composition characteristics across different seasons, focusing on April, June, and January varied in Chihuahua, Mexico [35]. Waste generation in January, corresponding to the low-temperature season, was found to be 28% lower than in April [15].

The analysis of waste generation across four sub-districts in Indonesia during both dry and rainy periods indicates a difference in generation rates. Specifically, the findings reveal that waste generation is higher during the rainy season compared to the dry season. However, the Jembrana sub-district stands out as an exception, exhibiting greater waste generation during the dry season. This analysis is outlined in detail in Table 5. Furthermore, upon conducting a thorough analysis of waste generation statistical difference using one-way ANOVA, it becomes evident that significant differences are observed solely in Muncar sub-district. During the sampling period, the communities experienced fruit harvest season, leading to a significant increase in waste generation. The elevated waste levels were primarily due to large quantities of organic wastes from fruit harvesting, processing, and consumption. This seasonal surge in waste contrasts with other periods of the year, reflecting a unique and heightened waste management challenge during the fruit harvest season.

**Table 5:** Analysis of statistical difference using one-way ANOVA

Samples	Season			Number of samples
	Rainy season	Dry season	Average	
Banyuwangi	0.4033	0.3558	0.3795	50
Muncar	0.4996*	0.3435*	0.4215	46
Jembrana	0.4829	0.5545	0.5187	28
Negara	0.5432	0.4729	0.5080	37

^*^There is a significant difference in seasons in ANOVA statistical analysis complemented with Tukey’s post hoc analysis (using confidence levels 95% and 99%)

Although the change in seasons may not have a significant overall impact—since most households dispose of their waste in plastic bags, minimizing the influence of moisture on waste weight—it's important to note that one out of eight sampling activities (12.5%) shows a statistically significant difference. While this may appear limited, such a finding cannot be overlooked as it highlights that seasons may influence waste characteristics under certain conditions. This underscores the need for continued monitoring and nuanced interpretation of seasonal variations in waste data.

## Conclusions

Based on these analyses, waste generation is unlikely to vary significantly whether sampled over four or eight days, both on weekdays and weekends. Additionally, the variation in waste composition showed no substantial differences, with only one out of eight sampling activities (12.5%) exhibiting a statistically significant deviation. While this proportion may seem minor, it is meaningful and should not be overlooked as it indicates that under certain local conditions, temporal factors can influence waste characteristics. This outcome may be related to the fact that sampling did not take place in major cities or metropolitan areas; instead, it was conducted in regions with predominantly rural or semi-urban characteristics, where daily activities remain relatively constant across the week. This is likely due to a high proportion of populations being engaged in non-office-based occupations. Furthermore, the analysis of waste generation across four Indonesian sub-districts revealed significant differences only between dry and rainy periods in Muncar sub-district. However, seasonal changes have little impact on overall waste generation as most households use plastic bags to cover waste before putting it into waste bins for disposal, maintaining a consistent overall weight. For future research, expanding the sampling to include metropolitan areas, a broader range of occupational contexts, and longer-term seasonal tracking will provide a more comprehensive understanding dynamics of waste generation and how it will affect sampling duration.

## Supplementary Information

Below is the link to the electronic supplementary material.

Supplementary file 1.

Supplementary file 2.
