# Supplementary material for: Implications of shorter sampling durations on the analysis of municipal solid waste generation and composition
Source: J Mater Cycles Waste Manag. 2025 Oct 30;28(1):133–40. doi: 10.1007/s10163-025-02401-6 (PMC12789102; doi:10.1007/s10163-025-02401-6)
Supplement: Supplementary file 1 [file 10163_2025_2401_MOESM1_ESM.docx]

**Assessing the Implications of Reduced Sampling Durations on the Analysis of Municipal Solid Waste Generation**

Emenda Sembiring^1*^, Attar Hikmahtiar Ramadan^1^, Muh. Farid^1^

^1^Air and Waste Management Research Group, Faculty of Civil and Environmental Engineering, Institut Teknologi Bandung, Bandung 40132, Indonesia

Corresponding Author: [emenda@itb.ac.id](mailto:emenda@itb.ac.id)

S1. Boxplot Comparison of Waste Generation

| **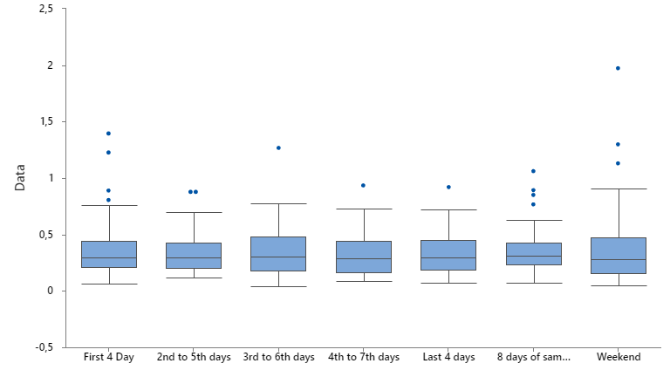** | **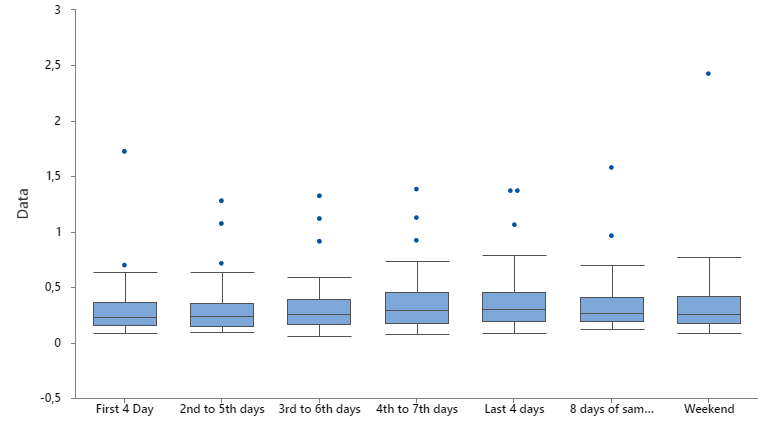** |
| --- | --- |
| **Banyuwangi Dry Season** | **Muncar Dry Season** |
| **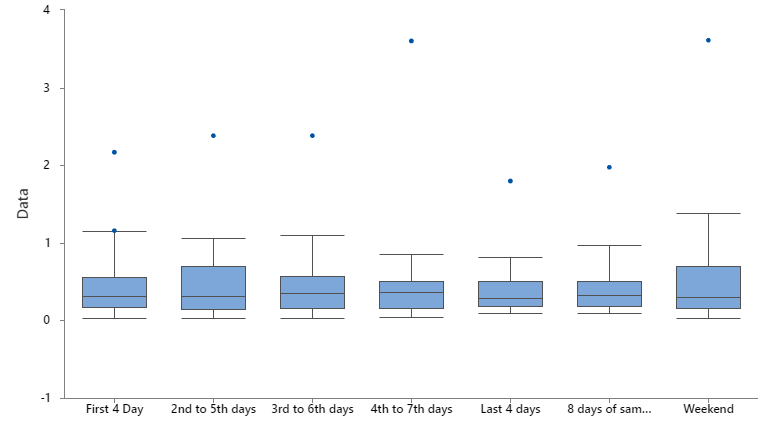** | **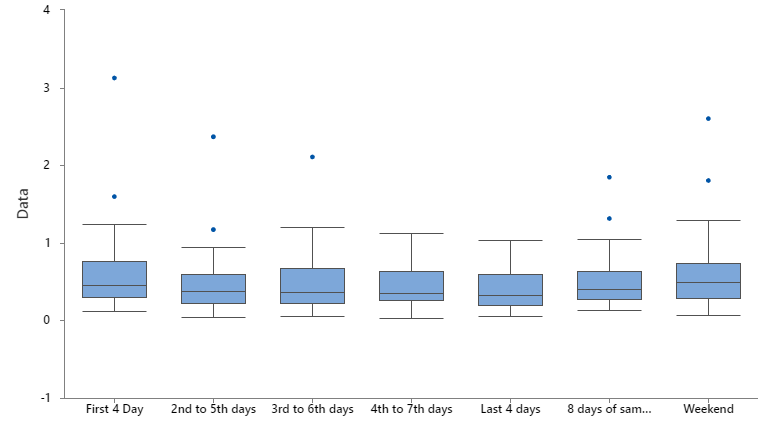** |
| **Banyuwangi Rainy Season** | **Muncar Rainy Season** |
| **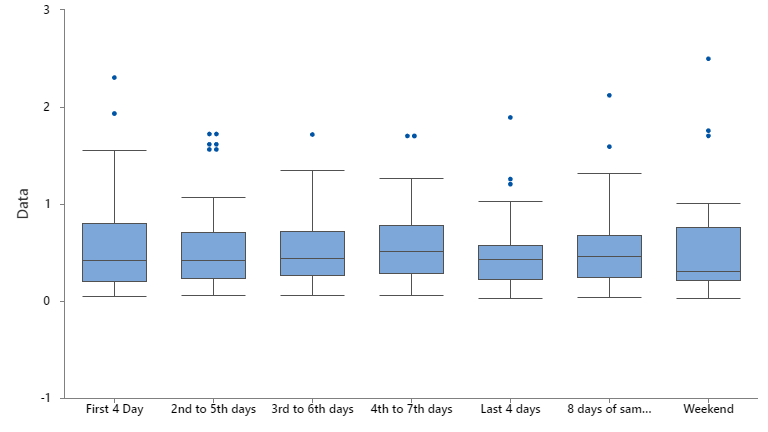** | **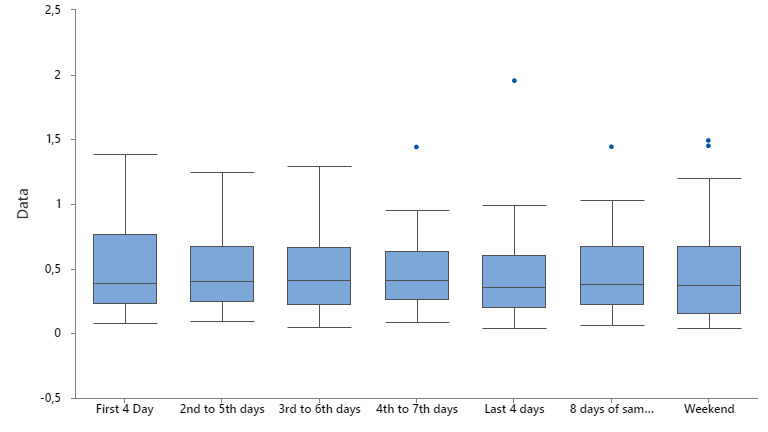** |
| **Jembrana Dry Season** | **Negara Dry Season** |
| **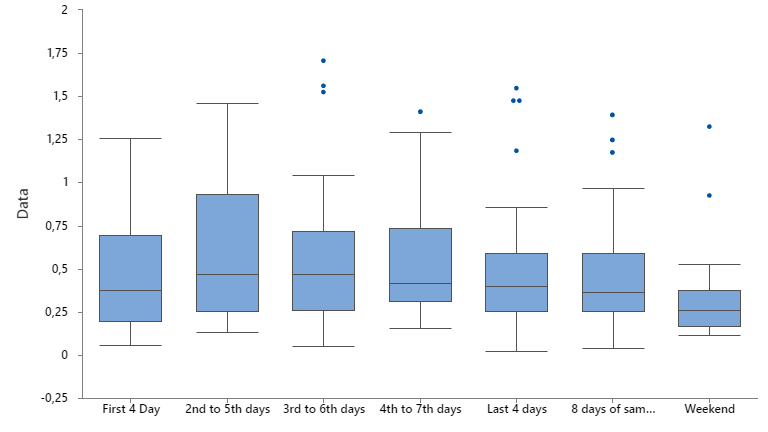** | **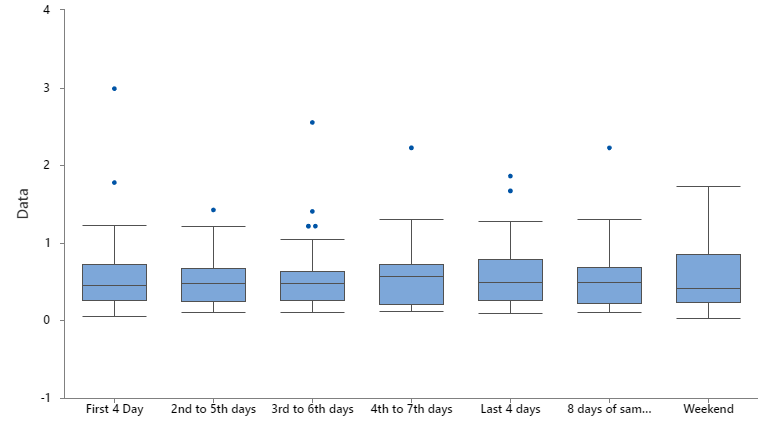** |
| **Jembrana Rainy Season** | **Negara Rainy Season** |

**Suplementary 1.** Boxplot of the sampling result analysis

S2. Bar Chart of Waste Composition

| 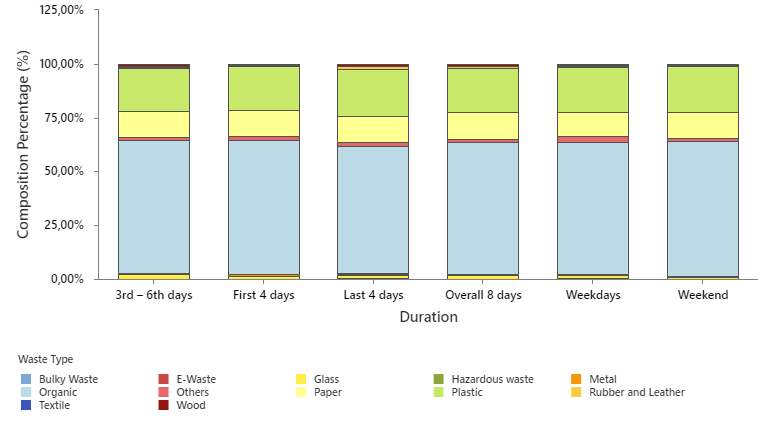 | **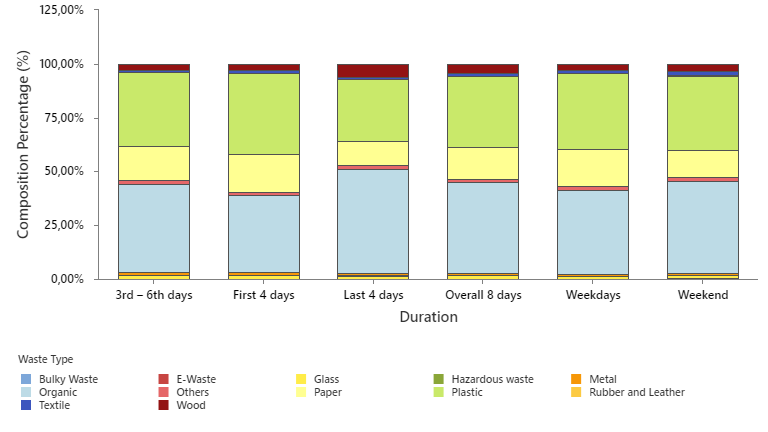** |
| --- | --- |
| **Banyuwangi Dry Season** | **Muncar Dry Season** |
| **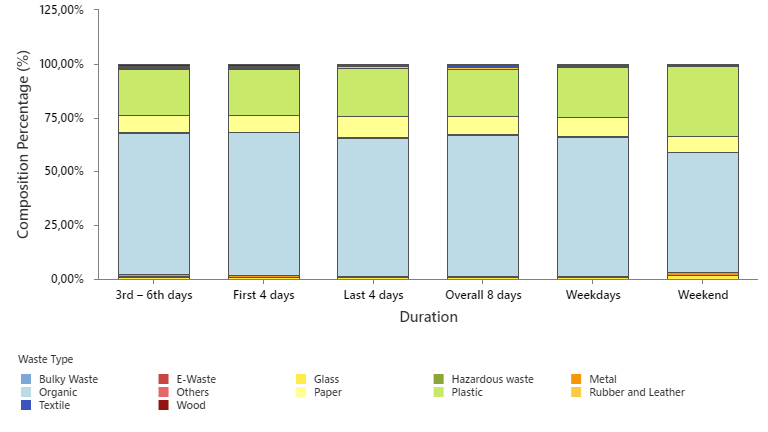** | **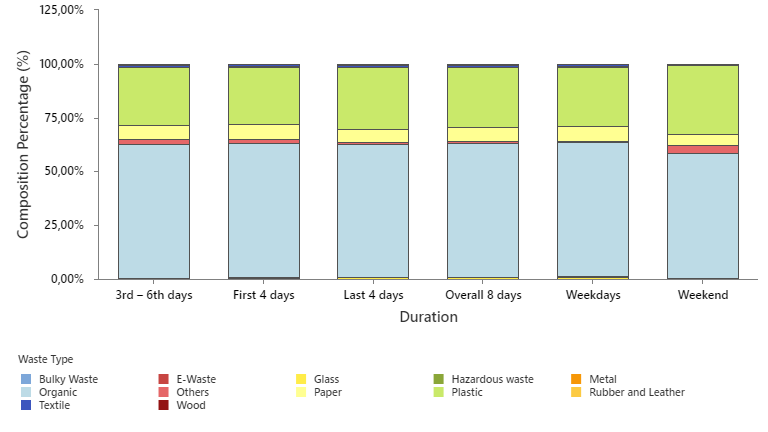** |
| **Banyuwangi Rainy Season** | **Muncar Rainy Season** |
| **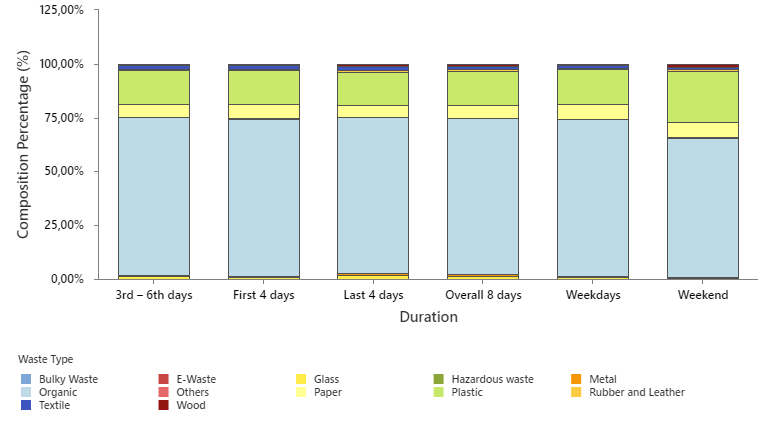** | **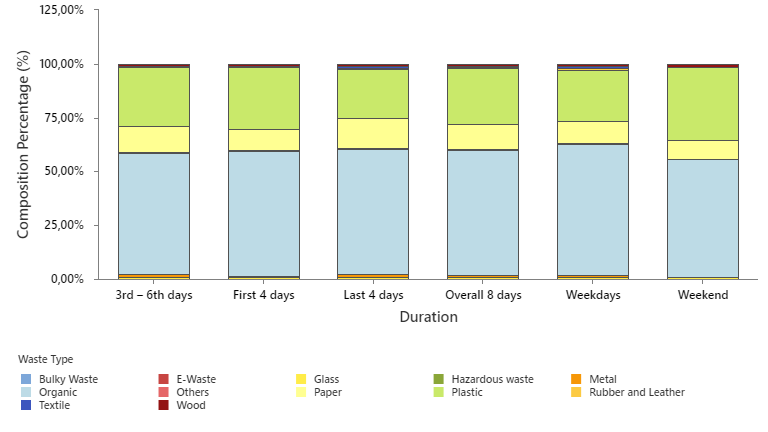** |
| **Jembrana Dry Season** | **Negara Dry Season** |
| **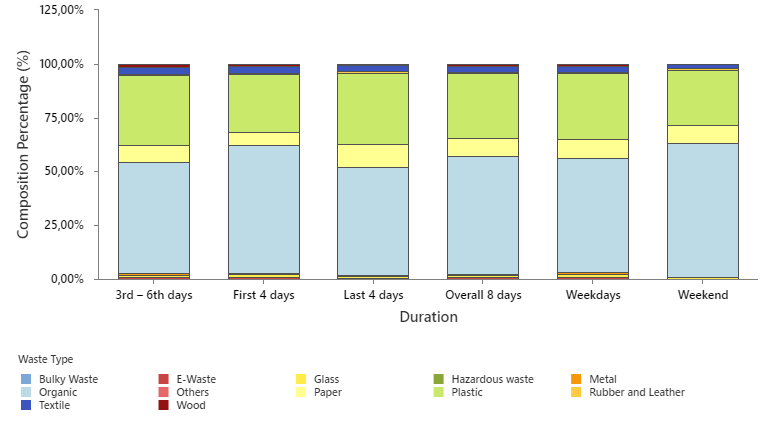** | **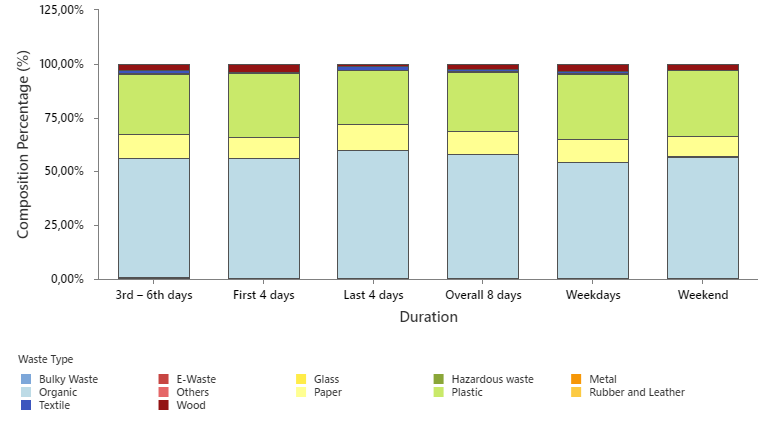** |
| **Jembrana Rainy Season** | **Negara Rainy Season** |

**Suplementary 2.** Bar chart of Waste Composition in each location

S3. Table of Waste Composition

1. **Solid Waste Composition**
2. **Dry Season**

| **Banyuwangi** | | | | |
| --- | --- | --- | --- | --- |
| **Category** | **High Income** | **Middle Income** | **Low Income** | **Average** |
| Organic | 61.44% | 61.11% | 61.31% | 61.29% |
| Wood | 0.46% | 1.16% | 0.47% | 0.70% |
| Paper | 11.71% | 11.72% | 13.17% | 12.20% |
| Metal | 0.38% | 0.57% | 1.16% | 0.70% |
| Rubber and Leather | 1.21% | 0.26% | 0.65% | 0.70% |
| Textile | 0.74% | 0.36% | 0.35% | 0.48% |
| Glass | 1.19% | 1.96% | 1.08% | 1.41% |
| Plastic | 20.57% | 21.55% | 19.64% | 20.59% |
| Hazardous waste | 0.00% | 0.08% | 0.10% | 0.06% |
| E-Waste | 0.09% | 0.02% | 0.50% | 0.20% |
| Bulky Waste | 0.00% | 0.06% | 0.00% | 0.02% |
| Others | 2.20% | 1.12% | 1.57% | 1.63% |

| **Muncar** | | | | |
| --- | --- | --- | --- | --- |
| **Category** | **High Income** | **Middle Income** | **Low Income** | **Average** |
| Organic | 45.82% | 41.49% | 38.49% | 41.93% |
| Wood | 5.41% | 4.27% | 2.93% | 4.20% |
| Paper | 13.29% | 16.15% | 15.78% | 15.07% |
| Metal | 0.55% | 1.46% | 0.55% | 0.86% |
| Rubber and Leather | 0.12% | 0.06% | 0.15% | 0.11% |
| Textile | 0.66% | 1.13% | 2.53% | 1.44% |
| Glass | 1.09% | 1.92% | 0.95% | 1.32% |
| Plastic | 32.41% | 32.69% | 35.34% | 30.56% |
| Hazardous waste | 0.00% | 0.00% | 0.14% | 0.05% |
| E-Waste | 0.02% | 0.03% | 0.24% | 0.10% |
| Bulky Waste | 0.06% | 0.10% | 0.00% | 0.06% |
| Others | 0.57% | 0.69% | 2.90% | 1.39% |

| **Jembrana** | | | | |
| --- | --- | --- | --- | --- |
| **Category** | **High Income** | **Middle Income** | **Low Income** | **Average** |
| Organic | 72.07% | 74.62% | 71.31% | 72.66% |
| Wood | 0.33% | 0.40% | 1.15% | 0.62% |
| Paper | 7.55% | 6.20% | 8.16% | 7.30% |
| Metal | 0.20% | 0.65% | 0.30% | 0.38% |
| Rubber and Leather | 0.71% | 0.58% | 0.48% | 0.59% |
| Textile | 0.81% | 1.41% | 2.72% | 1.65% |
| Glass | 0.67% | 0.90% | 2.34% | 1.30% |
| Plastic | 17.62% | 15.04% | 13.44% | 15.37% |
| Hazardous waste | 0.01% | 0.02% | 0.00% | 0.01% |
| E-Waste | 0.00% | 0.02% | 0.01% | 0.01% |
| Bulky Waste | 0.00% | 0.01% | 0.00% | 0.00% |
| Others | 0.05% | 0.17% | 0.09% | 0.10% |

| **Negara** | | | | |
| --- | --- | --- | --- | --- |
| **Category** | **High Income** | **Middle Income** | **Low Income** | **Average** |
| Organic | 64.48% | 57.73% | 61.14% | 61.12% |
| Wood | 0.00% | 1.77% | 0.00% | 0.59% |
| Paper | 9.57% | 9.69% | 13.50% | 10.92% |
| Metal | 2.30% | 0.45% | 0.21% | 0.99% |
| Rubber and Leather | 0.11% | 0.70% | 0.70% | 0.50% |
| Textile | 0.34% | 0.87% | 0.33% | 0.51% |
| Glass | 0.52% | 0.50% | 1.53% | 0.85% |
| Plastic | 22.55% | 27.96% | 21.64% | 24.05% |
| Hazardous waste | 0.00% | 0.05% | 0.00% | 0.02% |
| E-Waste | 0.00% | 0.04% | 0.00% | 0.01% |
| Bulky Waste | 0.00% | 0.03% | 0.00% | 0.01% |
| Others | 0.12% | 0.22% | 0.96% | 0.43% |

1. **Rainy Season**

| **Banyuwangi** | | | | |
| --- | --- | --- | --- | --- |
| **Category** | **High Income** | **Middle Income** | **Low Income** | **Average** |
| Organic | 65.95% | 74.97% | 67.74% | 69.56% |
| Wood | 0.49% | 0.17% | 0.03% | 0.23% |
| Paper | 8.70% | 3.25% | 8.92% | 6.96% |
| Metal | 0.75% | 2.19% | 0.24% | 1.06% |
| Rubber and Leather | 1.40% | 0.00% | 0.46% | 0.62% |
| Textile | 0.18% | 0.00% | 0.15% | 0.11% |
| Glass | 0.90% | 0.00% | 3.46% | 1.45% |
| Plastic | 20.22% | 19.41% | 18.20% | 19.28% |
| Hazardous waste | 0.00% | 0.00% | 0.00% | 0.00% |
| E-Waste | 0.00% | 0.00% | 0.00% | 0.00% |
| Bulky Waste | 0.00% | 0.00% | 0.00% | 0.00% |
| Others | 1.40% | 0.00% | 0.81% | 0.74% |

| **Muncar** | | | | |
| --- | --- | --- | --- | --- |
| **Category** | **High Income** | **Middle Income** | **Low Income** | **Average** |
| Organic | 57.70% | 65.17% | 69.29% | 64.05% |
| Wood | 0.32% | 0.22% | 0.28% | 0.27% |
| Paper | 10.74% | 6.73% | 4.18% | 7.22% |
| Metal | 0.29% | 0.34% | 0.16% | 0.27% |
| Rubber and Leather | 0.20% | 0.13% | 0.36% | 0.23% |
| Textile | 0.28% | 0.80% | 1.40% | 0.83% |
| Glass | 2.72% | 0.25% | 0.07% | 1.01% |
| Plastic | 25.77% | 27.18% | 24.04% | 25.66% |
| Hazardous waste | 0.00% | 0.00% | 0.00% | 0.00% |
| E-Waste | 0.09% | 0.15% | 0.21% | 0.15% |
| Bulky Waste | 0.04% | 0.00% | 0.00% | 0.01% |
| Others | 2.07% | 1.35% | 0.09% | 1.17% |

| **Jembrana** | | | | |
| --- | --- | --- | --- | --- |
| **Category** | **High Income** | **Middle Income** | **Low Income** | **Average** |
| Organic | 24.32% | 46.23% | 34.24% | 34.93% |
| Wood | 0.01% | 1.23% | 0.63% | 0.62% |
| Paper | 8.17% | 6.64% | 9.85% | 8.22% |
| Metal | 0.17% | 0.42% | 0.92% | 0.50% |
| Rubber and Leather | 0.18% | 0.73% | 0.60% | 0.50% |
| Textile | 4.67% | 3.00% | 1.76% | 3.14% |
| Glass | 6.58% | 0.26% | 0.25% | 2.36% |
| Plastic | 33.94% | 34.53% | 27.80% | 32.09% |
| Hazardous waste | 0.26% | 0.00% | 0.09% | 0.12% |
| E-Waste | 0.00% | 0.41% | 0.86% | 0.42% |
| Bulky Waste | 21.70% | 6.56% | 23.00% | 17.09% |
| Others | 0.00% | 0.00% | 0.00% | 0.00% |

| **Negara** | | | | |
| --- | --- | --- | --- | --- |
| **Category** | **High Income** | **Middle Income** | **Low Income** | **Average** |
| Organic | 30.42% | 40.31% | 44.02% | 38.25% |
| Wood | 8.18% | 1.88% | 1.20% | 3.75% |
| Paper | 6.81% | 10.54% | 12.69% | 10.01% |
| Metal | 0.04% | 0.13% | 0.42% | 0.20% |
| Rubber and Leather | 0.04% | 0.32% | 0.21% | 0.19% |
| Textile | 1.50% | 0.91% | 0.57% | 0.99% |
| Glass | 0.02% | 0.11% | 0.37% | 0.17% |
| Plastic | 24.22% | 28.04% | 28.22% | 26.83% |
| Hazardous waste | 0.00% | 0.05% | 0.00% | 0.02% |
| E-Waste | 0.01% | 0.13% | 0.15% | 0.10% |
| Bulky Waste | 28.76% | 17.52% | 11.90% | 19.39% |
| Others | 0.00% | 0.06% | 0.25% | 0.10% |
